# Safety and Effectiveness of Bone Marrow Cell Concentrate in the Treatment of Chronic Critical Limb Ischemia Utilizing a Rapid Point-of-Care System

**DOI:** 10.1155/2017/4137626

**Published:** 2017-01-17

**Authors:** Venkatesh Ponemone, Saniya Gupta, Dalip Sethi, Manish Suthar, Monika Sharma, Richard J. Powell, Kenneth Lee Harris, Nungshi Jungla, Priyadarshini Arambam, Upendra Kaul, Ashok Seth, Suhail Bukhari

**Affiliations:** ^1^TotipotentRX Centre for Cellular Medicine, Subsidiary of Cesca Therapeutics Inc., Gurgaon, India; ^2^Geisel School of Medicine, Dartmouth-Hitchcock Medical Centre, Lebanon, NH, USA; ^3^Fortis Escorts Heart Institute and Research Centre, New Delhi, India

## Abstract

Critical limb ischemia (CLI) is the end stage of lower extremity peripheral vascular disease (PVD) in which severe obstruction of blood flow results in ischemic rest pain, ulcers and/or gangrene, and a significant risk of limb loss. This open-label, single-arm feasibility study evaluated the safety and therapeutic effectiveness of autologous bone marrow cell (aBMC) concentrate in revascularization of CLI patients utilizing a rapid point-of-care device. Seventeen (17) no-option CLI patients with ischemic rest pain were enrolled in the study. Single dose of aBMC, prepared utilizing an intraoperative point-of-care device, the Res-Q™ 60 BMC system, was injected intramuscularly into the afflicted limb and patients were followed up at regular intervals for 12 months. A statistically significant improvement in Ankle Brachial Index (ABI), Transcutaneous Oxygen Pressure (TcPO_2_), mean rest pain and intermittent claudication pain scores, wound/ ulcer healing, and 6-minute walking distance was observed following aBMC treatment. Major amputation-free survival (mAFS) rate and amputation-free rates (AFR) at 12 months were 70.6% and 82.3%, respectively. In conclusion, aBMC injections were well tolerated with improved tissue perfusion, confirming the safety, feasibility, and preliminary effectiveness of aBMC treatment in CLI patients.

## 1. Introduction

Critical limb ischemia (CLI) is a debilitating cardiovascular disease characterized by severely impaired hemodynamic, chronic ischemic rest pain, ischemic ulcers and/or gangrene, and tissue loss. The incidence of critical limb ischemia is estimated to be approximately 500 to 1000 new cases per million people per year in the western countries and with an aging population, the risk is expected to increase (nearly double) due to persistent rates of tobacco use, an increase in type 2 diabetes, obesity, and sedentary lifestyle [1, 2]. Also, the incidences of cardiovascular events increase considerably among the afflicted patients. The US prevalence of CLI is currently 2.8 to 3.5 million people and is forecasted to grow between 3.6 and 4.5 million people by 2020 [3].

CLI is a severe obstruction of the arteries that causes significant decrease in the blood flow to the lower extremities. Chronic lack of blood supply results in a cascade of pathophysiological events that ultimately lead to severe rest pain in the foot or toes [4] and consequently progress to nonhealing ulcers and wounds on the leg, foot, or ankle at a later stage. Risk factors associated with the development of CLI include age, nicotine use, diabetes mellitus, hypertension, hypercholesterolemia, and family history of cardiovascular disease [5–8], driving our societal need to find novel clinical interventions which can restore seminormal limb function or at least stabilize disease progression.

The current mainstay treatment option for these patients is either surgical and/or endovascular procedures, aimed at revascularization and improving perfusion to the distal limb [3]. Despite technical advances, more than 30% of patients do not qualify as candidates for revascularization because of excessive surgical risk and/or adverse vascular involvement [9]. The other treatment options for ischemic limb include pharmacological approaches using lipid reduction, antiplatelet, and antihypertensive therapies [10] or nonpharmacological approaches for restoration of perfusion and local wound care, which include pressure off-loading, infection control, and meticulous debridement as needed [11]. However, no pharmacologic therapy has proven effective in reducing amputation rates in CLI patients [12]. The last available option for these patients who have exhausted their surgical options is limb amputation [13, 14]. It is estimated that the mortality rate in these patients who are not eligible for surgical revascularization or endovascular treatment, designated as “no-option” CLI patients, within 6 months from diagnosis is approximately 20%, and at 1 year it ranges from 10 to 40%, while another 40% would undergo major limb amputation [1, 15, 16]. Also, it is projected that 208,000 lower limb amputations related to CLI are performed in USA every year with more than 65,000 being major limb amputations. The unfortunate reality is that only 15% of the amputated patients lead a normal life with a proper prosthetic limb, while the remaining 85% of the amputees are unable to have adequate ambulation to care for themselves again [17, 18].

Therefore, this is a critical, unmet medical need where innovative approaches to augment vascular regeneration are urgently needed to save lives and limbs and improve overall quality of life. Cell-based regenerative therapies aiming at neovascularization and improved limb perfusion have been proposed as novel treatment strategies. In 1997, Asahara et al. and Shi et al. identified a class of bone marrow derived circulating endothelial progenitor cells (EPCs) that contribute to angiogenesis and/or vasculogenesis in ischemic tissues and improved tissue perfusion [19, 20]. A small first-in-man clinical trial, reported by Tateishi-Yuyama et al., showed safety and promising effects of autologous bone marrow- (BM-) derived cell therapy in patients with critical limb ischemia [21]. Since then, research focusing on the use of autologous cell therapy has shown promising results and has led to several clinical studies in the last 10 years, wherein autologous bone marrow cell (aBMC) implantation in patients with CLI who had no other alternative treatment options showed safety and efficacy of this therapy based on statistical analysis, providing new hope for CLI patients [12, 14, 22–28]. These studies have also reported the capacity of bone marrow stem and progenitor cells to promote revascularization, thereby improving limb perfusion sufficiently to resolve pain at rest and resulting in limb salvage.

Bone marrow cell concentrate (BMC) functions via different mechanisms; however, it is not clear which subset of BMC is optimal for treating CLI [28, 29]. Bone marrow cell concentrate contains a heterogeneous population of endothelial progenitor cells, mesenchymal stem cells, and hematopoietic stem cells that stimulate angiogenesis to treat disorders of inadequate tissue perfusion [30]. Angiogenesis occurs in stages through which migrated endothelial cells undergo tube formation and remodelling of newly formed vessels into 3-dimensional networks with regression of unnecessary microvessels [31]. The transplanted bone marrow cells migrate to the ischemic endothelial surface, where they improve the density of capillaries and microvessels primarily by secreting growth factors and cytokines (like Vascular Endothelial Growth Factor (VEGF), Transforming Growth Factor-*β* (TGF-*β*), and Fibroblast Growth Factor (FGF)), which exhibit paracrine effect and ameliorate the blockage of arteries. In addition, the secreted cytokines regulate the microenvironment, thereby relieving symptoms through their immune-suppressive character promoted by CD14^+^ monocytes [18, 28]. Although the exact mechanism of neovascularization remains uncertain, an increasing number of clinical trials using bone marrow derived progenitor cells have demonstrated clinical benefit, showing improvement in objective and subjective measures of perfusion, pain reduction, increase in total walking distance, and decreased rate of amputation [3, 28]. For all of these reasons, cell-based therapy holds promise as potential novel therapeutic modality for patients with advanced PVD.

The aim of this open-label, nonrandomized, single-centre, feasibility study was to evaluate the safety and feasibility of a rapid point-of-care device, Res-Q 60 BMC system, in successfully processing autologous bone marrow (BM) and the preliminary therapeutic effectiveness of BM cellular therapy in no-option CLI patients.

## 2. Materials and Methods

### 2.1. Study Design

The critical limb ischemia-Stem Cell Therapy (CLI-STEM) study was designed as an open-label, prospective, single-arm, single-centre, phase Ib/feasibility study. A total of 17 patients were enrolled in the study who were suffering from end-stage CLI with ischemic rest pain and minor tissue loss (thirteen (13) patients with atherosclerotic PAD and four (4) patients with Buerger's disease) in whom all previous therapeutic revascularization strategies had failed (“no-option patients”). In addition, these patients demonstrated infrainguinal atherosclerosis with stenosis (>70%) or occlusion (100%) of a major vessel, having an Ankle Brachial Index (ABI) ≤ 0.6 and Transcutaneous Oxygen Pressure (TcPO_2_) < 35 mmHg. The diagnosis of limb ischemia was confirmed by CT Angiography (CTA) and amputation was the only treatment option available as determined and confirmed by vascular surgeon and interventional radiologist. All the enrolled patients received single dose of intramuscular injections of autologous bone marrow cell (aBMC) concentrate along with standard-of-care (conventional therapy) medications and medical management.

The protocol was approved by “The Independent Ethics Committee (TIEC)” (TIEC/2010/30/04) and the “Institutional Committee for Stem Cell Research and Therapy (IC-SCRT)” of the participating institution and registered on clinicaltrials.gov (Identifier: NCT01472289). The study was conducted in accordance with the Declaration of Helsinki. Written informed consent for participation was obtained from all the patients.

### 2.2. Inclusion Criteria

Eligible patients were men and women 18 to 65 years of age with atherosclerotic Ischemic Peripheral Arterial Disease (PAD) or Thromboangiitis Obliterans (TAO)/Buerger's disease of the lower extremities defined as ischemic pain at rest and minor tissue loss and ischemic ulcers or gangrene, which may be dry or humid, with Ankle Brachial Index (ABI) ≤0.6 or ankle pressure ≤60 mmHg or TcPO_2_ ≤35 mmHg in the foot, without acceptable options for revascularization. Patients considered for near-term limb amputations (above the ankle) due to severe life threatening ischemia were required to establish controlled blood sugar levels (HbA1c < 8%), controlled blood pressure with antihypertensive therapy as necessary, and control of hyperlipidaemia with statins and antiplatelet therapy prior to entry.

### 2.3. Exclusion Criteria

Main exclusion criteria were HbA1c > 8.0%; serum creatinine ≥ 2.0 mg/dL and serum total bilirubin ≥ 2.0 mg/dL; known CLI patients with failed ipsilateral revascularization procedure or requiring amputation proximal to transmetatarsal level; moderate to severe Chronic Obstructive Pulmonary Disease (COPD) with GOLD classification of IIb or III; uncontrolled Congestive Heart Failure (CHF) or left ventricular ejection fraction <25%; stroke or myocardial infarction within last 3 months; serious and/or unstable infection of the involved extremity; documented terminal illness or cancer with life expectancy less than 1 year; pregnant or lactating women or patients exhibiting spreading (wet) gangrene; and prior enrollment in another investigational trial or completing one within the last 3 months.

### 2.4. Treatment Procedure

The treatment procedure involved aspiration and processing of bone marrow and intramuscular injections of autologous bone marrow cell concentrate into the study limb using an investigational device the SurgWerks-CLI Kit (Cesca Therapeutics, Inc., USA) containing the Res-Q 60 BMC system (point-of-care technology, License #MD-826, Central Drug Standard Control Organisation, India). This point-of-care kit is inclusive of the devices required to process and deliver the aBMC, specifically aiding in the processing of cells (i.e., rapid purification/concentration) at the patients' bed side, with a single sitting in the operation theatre. Procedure was carried out under conscious sedation and strict aseptic conditions. A total volume of 120 mL of uncoagulated bone marrow was aspirated from multiple sites from the posterior superior iliac crest in heparinized syringes under local anaesthesia using an 11-gauge 5-side-hole Jamshidi needle. A small aliquot of 1 mL was collected from the aspirated bone marrow and sample was analysed for cell counts, sterility, and viability.

The harvested bone marrow was processed and concentrated using the Res-Q 60 BMC system (Thermogenesis Corp.). This point-of-care system is an automated cell processing medical device that concentrates the bone marrow by a density gradient centrifugation method. The aspirated bone marrow was transferred aseptically and equally into two Res-Q 60 BMC devices using a clot filter (included in the SurgWerks-CLI Kit) to remove any large particulate matter, such as clots, fat, and bone chips. The processing was carried out in the operating room at the patient's bed side and 24 mL (12 mL from each device) of BMC enriched in progenitor cells was collected from the devices. Aliquots (4 mL) of aBMC were collected from the processed sample and later analysed for cell counts, sterility, potency, and viability.

The remaining 20 mL autologous bone marrow concentrate was intramuscularly injected in 0.5 mL volumes per injection site (total of 40 sites, 1.5 cm depth) in the gastrocnemius/calf muscle of the ischemic limb with a 3 × 3 cm grid using a 22-gauge needle. In case of presence of wound/ulcer, 4–6 injections of 0.25 mL each (max 1.5 mL) were administered around the wound periphery subsequent to wound debridement as required.

Patients were discharged on the first postoperative day, scheduled for follow-up at 1, 3, 6, and 12 months following cell therapy, and given standard-of-care medications, including aspirin/clopidogrel, ACE inhibitors, beta blockers and statins, and local wound therapy (systemic antibiotic therapy and manual debridement of necrotic tissue). Evaluation included subjective and objective measures of clinical, hemodynamic, and functional outcomes. Clinical outcomes included amputation status, rest pain, and intermittent claudication pain scores using visual analogue scale, 6-minute walking distance, and examination of ulcers/wound or gangrene. Hemodynamic outcome was evaluated by changes in Ankle Brachial Index (ABI) and Transcutaneous Oxygen Pressure (TcPO_2_). Functional outcome was evaluated using the SF-36 Quality of Life Questionnaire. CT Angiography was performed at baseline and repeated at 12 months for the qualitative and quantitative assessment of the degree of angiogenesis. Laboratory monitoring of haematology and blood chemistry was also performed.

### 2.5. Endpoints

The primary endpoint of this clinical study was to determine the safety of intramuscular administration of concentrated autologous BMC harvested, processed, and delivered using Res-Q 60 BMC system (SurgWerks-CLI kit), a rapid intraoperative point-of-care device in nonreconstructable CLI patients. While, the secondary endpoints were to assess preliminary effectiveness by measuring changes in blood perfusion parameters measured by ABI and TcPO_2_, changes in rest pain and intermittent claudication pain score, 6-minute walking distance, clinical examination of ulcer, gangrene, or wound healing, amputation-free survival rate at 12 months, and degree of angiogenesis both quantitative and qualitative measured using CT Angiography.

### 2.6. Statistical Analysis

All the statistical analysis for the data was done using the software SAS (Statistical Analysis System) version 9.1.3. All safety endpoints were analysed on intent-to-treat (ITT) population and all the primary and secondary efficacy endpoints were analysed as per-protocol (PP) population. Descriptive analysis was carried out, clinical event rates are presented in number and percentage (%), and continuous variables are presented as mean ± standard deviation. Intraindividual comparison of continuous variables at baseline with those at follow-up was performed with the paired* t*-test for normally distributed variables and Wilcoxon signed rank test for nonnormally distributed continuous variables. All reported adverse events (AEs) are regarded as severe adverse events (SAEs) and summarized using number (*n*) and percentage (%). All statistical tests were done using two-sided, 0.05 level of significance.

## 3. Results

### 3.1. Baseline Characteristics

**Figure 1:**
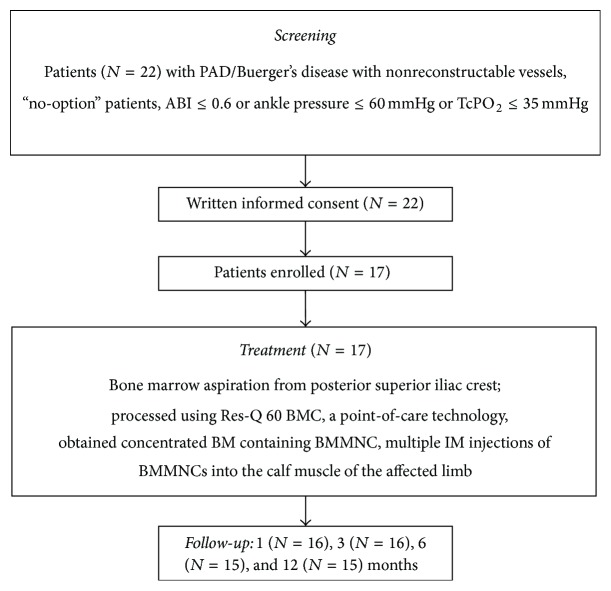
Study enrollment design.

The study enrollment diagram is shown in Figure 1, and the baseline clinical characteristics of the enrolled patients are summarized in Table 1. All the CLI patients screened had an underlying aetiology due to atherosclerotic arterial occlusive disease (PAD) or Thromboangiitis Obliterans (TAO)/ Buerger's disease. Out of the 22 patients screened, 17 patients met the eligibility criteria and were enrolled in the study. All patients were given intramuscular autologous BMC injections in their ischemic limb. The mean age of study patients was 48.8 ± 13.07 years. Of these 15 (88.2%) were male and 2 were (11.8%) female patients. Nine (52.9%) patients received treatment in their left limb, while eight (47.1%) received treatment in their right limb. Among the 17 patients who received aBMC treatment, 16 (94.1%) patients visited for posttreatment follow-up at 1 and 3 months, and 15 (88.2%) patients visited for follow-up at 6 and 12 months. All patients received antiplatelets, ACE (Angiotensin Converting Enzyme) inhibitors, and beta blocker drugs throughout the study period.

### 3.2. Safety and Feasibility

Bone marrow aspiration, processing, and injection were accomplished in the operating room in a single sitting in less than 60 min under local anaesthesia. Patients tolerated the procedure well, and transient smooth sedation was used to facilitate the bone marrow aspiration and injection procedure. There was no bleeding, infection, or procedure related complication, including local injection site swelling in all the subjects on the day of treatment after aBMC administration. The mean total nucleated cell count (TNCC) and mononuclear cell count (MNCC) in the 20 mL aBMC product injected into the afflicted limb were 8.04 × 10^8^ (±3.66) and 2.16 × 10^8^ (±1.02), respectively. The mean viable CD34^+^ cells were 4.06 × 10^6^ (±3.82) and the mean total colony forming units were 101.10 × 10^4^ (±113.61). The mean cell viability was found to be over 88.94%.  Table 2 outlines the mean cell product characterization pre- and postprocessing for bone marrow samples. Cell counts did not correlate with age, comorbidities, or outcome.

All the safety endpoints in the study were analysed on the ITT population. Out of 17 patients treated, adverse events were reported in seven (41.2%) patients, where three (17.6%) patients underwent major limb amputation (above the ankle), two (11.8%) patients underwent minor amputation (digit/s), and two unrelated deaths were reported (on day 121 and day 190 after treatment). All the reported adverse events were treated as serious adverse events in this study.

### 3.3. Preliminary Efficacy Endpoints

#### 3.3.1. Analysis of ABI and TcPO_2_

**Figure 2:**
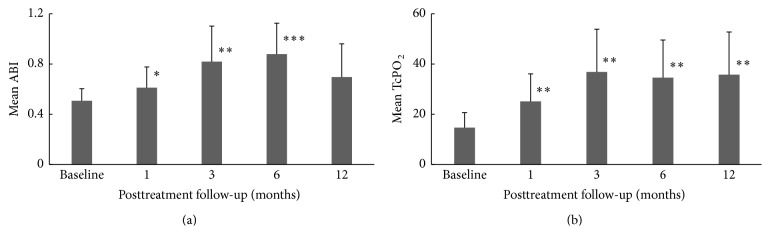
(a) Analysis of changes in ABI from baseline to 12 months (^*∗*^*p* < 0.05; ^*∗∗*^*p* < 0.01; ^*∗∗∗*^*p* < 0.001). (b) Analysis of changes in TcPO_2_ from baseline to 12 months (^*∗*^*p* < 0.05; ^*∗∗*^*p* < 0.01; ^*∗∗∗*^*p* < 0.001).

The efficacy endpoints were analysed on the per-protocol (PP) population basis (*N* = 14) and all patients showed improvement in their condition after intervention. ABI and TcPO_2_ were used as noninvasive techniques for assessing microvascular blood flow and tissue oxygen tension after autologous bone marrow concentrate intramuscular treatment. ABI was used to provide a measure of blood flow in the lower limbs and has a normal value of 0.91–1.3 [32], while TcPO_2_ was used to assess the partial pressure (tension) of oxygen in the capillaries of tissues of the lower limbs. TcPO_2_ values greater than 40 mmHg are said to be associated with good chances of healing while ≤40 mmHg is defined as low with poor chances of healing [32, 33]. Figures 2(a) and 2(b) show the changes in ABI and TcPO_2_ from baseline to 12 months after aBMC implantation, respectively.

**Figure 3:**
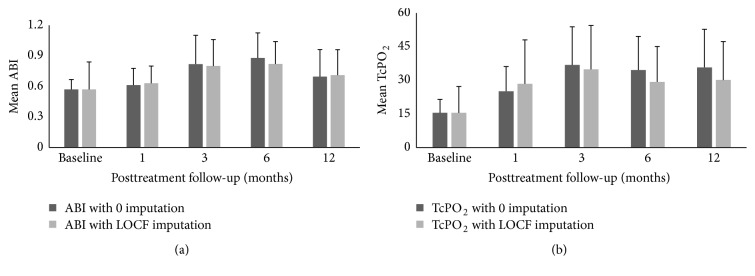
(a) Analysis of change in ABI using two imputation methods: “0” imputation and LOCF. (b) Analysis of change in TcPO_2_ using two imputation methods: “0” imputation and LOCF.

Analysis of the evaluable patients showed a statistically significant improvement in mean ABI post-BMC treatment from 1-month (*p* < 0.05) to 6-month (*p* < 0.001) follow-up period compared to baseline and gradually decreased from 6-month to 12-month follow-up period; however it remained higher than the baseline level. The BMC treated patients showed a statistically significant improvement in TcPO_2_ during the 12-month follow-up period (*p* < 0.01) as compared to baseline indicating an improved microvascular blood flow and tissue oxygen tension in the capillaries of the treated limb after intervention. The missing data were analysed by two commonly used imputation methods: method of “0” imputation (missing data is replaced by value zero) and last observation carried forward (LOCF) method (where a missing measurement of a participant is replaced by the participant's last observed value). The results indicate that no statistically significant difference in ABI and TcPO_2_ measurement was observed between these two methods. The mean ABI using the method of “0” imputation and LOCF method was 0.696 (±0.27) and 0.71 (±0.25) at 12 months posttreatment, indicating that there is no significant difference between the two imputation methods. Similarly, the mean TcPO_2_ value at 12 months posttreatment using the method of “0” imputation and LOCF method was 35.75 mmHg (±17) and 30.12 mmHg (±17.12), respectively, which was not a statistically significant difference. Figures 3(a) and 3(b) show the comparison between the changes in reading using the two imputation methods for ABI and TcPO_2_, respectively.

**Figure 4:**
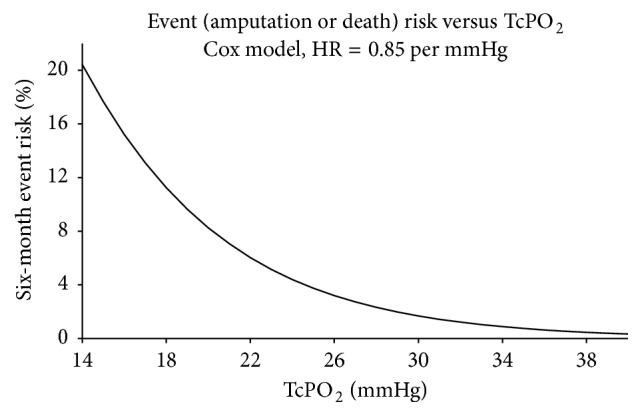
Correlation of risk of amputation versus TcPO_2_.

TcPO_2_ could also be used as a predictive marker for major limb amputation and literature confirming this correlation is quite strong. To make this assessment we used a Cox proportional hazard model to investigate the effect of TcPO_2_ on the time to event (amputation and death) using the study dataset points The model based results show that the event rate decreases by a factor of about 0.85 (HR = hazard; ratio = event rate ratio) on average for every 1 mmHg increase in TcPO_2_. The same 0.85 value is obtained using either baseline or 1-month TcPO_2_ reading. On an average the event rate decreases about 15% for every 1 mmHg increase in TcPO_2_, which is statistically significant (*p* < 0.05). Based on this, the model based relationship between TcPO_2_ and event risk was analysed and is plotted in Figure 4. At mean TcPO_2_ of 15 mmHg, the overall six-month event risk is about 18%; however if mean TcPO_2_ increases to 25 mmHg, the model based six-month event risk is less than 4% and if mean TcPO_2_ is 35 mmHg, the model based six-month risk is less than 1%. Thus improving TcPO_2_ correlates with reduced amputation/death events, with subjects having a baseline value of 15 mmHg having a much higher risk of an event versus patients with a baseline value of >25 mmHg. The data suggests that treatment improves TcPO_2_ which can safely predict that amputation “event” rates will decrease.

#### 3.3.2. Functional Improvement, Wound Healing, and Rest Pain

The efficacy endpoints were analysed on the per-protocol (PP) population basis (*N* = 14) and all patients showed improvement in the 6-minute walking distance, rest pain and intermittent claudication pain scores and healing of ulcers, and wound and gangrene after BMC intervention. 

**Figure 5:**
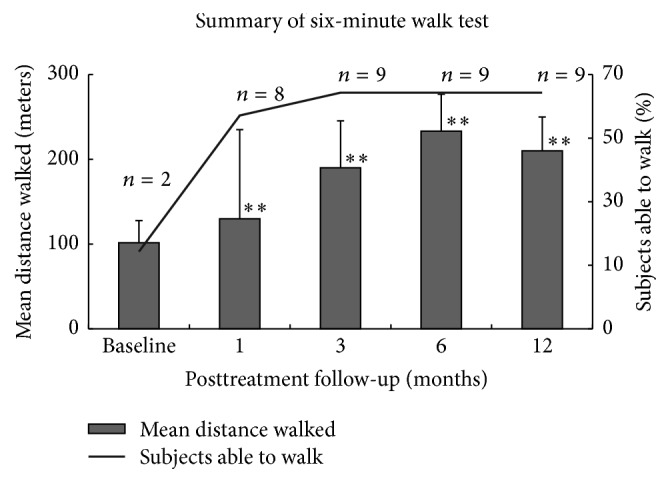
Analysis of 6-minute walking distance from baseline to 12 months (^*∗∗*^*p* < 0.01).

*6 Minute Walk Test*.  Figure 5 shows the distribution of 6-minute walk test from baseline to 12 months. At baseline, out of the 14 patients analysed, 2 (14.3%) patients were able to walk a mean distance of 101.5 meters in six minutes. Following BMC intervention, there was a significant improvement in the number of patients who were able to walk and the distance covered by them in 6 minutes. At 1-month follow-up 8 (57.1%) patients were able to walk a mean distance of 130.0 meters in 6 minutes and at 3-month follow-up, 9 (64.3%) patients were able to walk and covered a mean distance of 190.0 meters in 6 minutes. Thereafter the number of patients who were able to walk remained the same at 6- and 12-month posttreatment follow-up, but the mean distance covered by them increased to 233.3 meters and 210.0 meters, respectively. Overall, the change in number of patients able to walk and the mean distance walked from baseline to 12 months were statistically significant (*p* < 0.01). 

**Figure 6:**
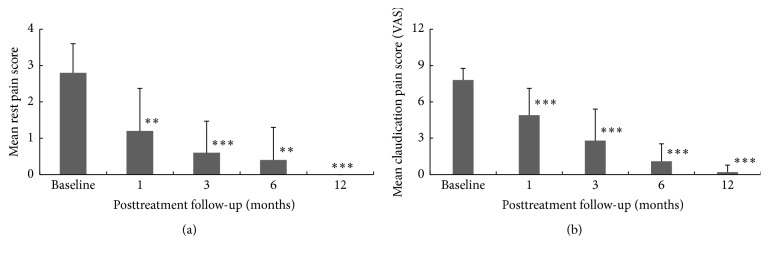
(a) Analysis of rest pain and changes from baseline to 12 months (^*∗∗*^*p* < 0.01; ^*∗∗∗*^*p* < 0.001). (b) Analysis of intermittent claudication pain score and changes from baseline to 12 months (^*∗∗*^*p* < 0.01; ^*∗∗∗*^*p* < 0.001).

*Rest Pain and Intermittent Claudication Pain Score*. Figures 6(a) and 6(b) show the change in rest pain and intermittent claudication pain score from baseline to 12-month after BMC treatment. Rest pain is defined as a burning sensation felt at rest, usually in the skin of the foot, and was assessed using the grading system (0–3) as defined in the protocol, that is, no pain = 0 while severe pain = 3. Intermittent claudication is a crampy leg pain that occurs during exercise, especially walking, and was measured using the Visual Analogue Scale (VAS) (0–10, with No Pain = 0 and Worst Pain = 10). The mean pain score at baseline for rest pain and intermittent claudication were 2.8 (±0.80) and 7.8 (±0.97), respectively. Following treatment with BMC, there was a time dependent, significant reduction in the rest pain and claudication pain score from 1-month through 12-month follow-up period. At 1 month post-BMC treatment, rest pain and intermittent claudication pain score reduced significantly to 1.2 (±1.17) (*p* < 0.01) and 4.9 (±2.22) (*p* < 0.001), respectively, and by the end of 12-month follow-up period, the BMC treated patients experienced no pain at rest (*p* < 0.001), while intermittent claudication pain score reduced significantly to 0.2 (±0.58) (*p* < 0.001). 

**Figure 7:**
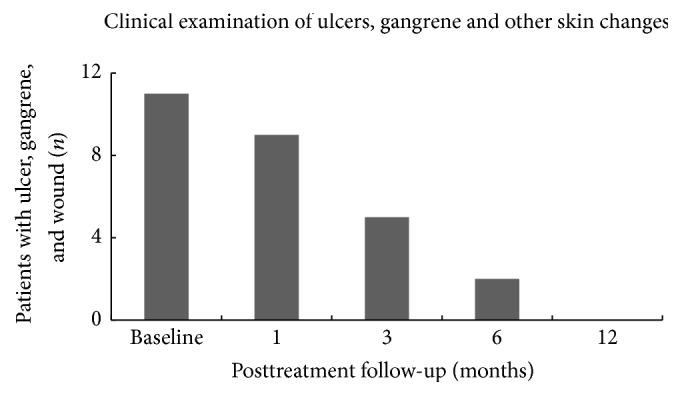
Summary of ulcer, gangrene, and wound healing.

*Clinical Examination of Ulcer, Gangrene, and Wounds*.  Figure 7 shows the summary of healing time for ulcers, gangrene, and wounds. The ulceration and gangrene in the affected limb of the patients were evaluated by visual clinical inspection by trained and well qualified medical doctors at various follow-up time intervals. At baseline, 11 (78.5%) of 14 subjects were presented with foot ischemic ulcers and/or gangrene. However, after receiving BMC treatment, it was observed at 12-month follow-up that all the patients who had reported ulcers, gangrene, or wound at baseline had a complete ulcer healing. Most of the patients with ischemic ulcers had ingrowth of granulation tissue indicating a significant wound/ulcer healing process.

### 3.4. Time to Amputation and Amputation Rates

**Figure 8:**
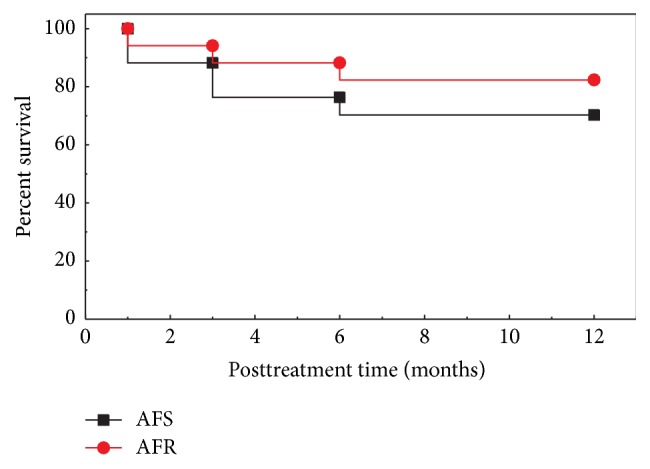
Kaplan-Meier analysis of major amputation-free survival and major amputation-free rates.

The median time to major amputation was 6 months in all the BMC treated patients. Major amputation-free survival rate and total amputation rates were analysed on the ITT population (*N* = 17). 12 out of 17 (70.6%) patients survived without any major limb amputation (mAFS: major amputation-free survival) and 14 out of 17 (82.3%) patients' limbs were salvaged from major limb amputation (AFR: major amputation-free rate). Kaplan-Meier Curves for major amputation-free survival and amputation-free rate are presented in Figure 8. All the events related to limb amputations were severe in nature and were due to the natural progression of the disease.

### 3.5. Degree of Angiogenesis

**Figure 9:**
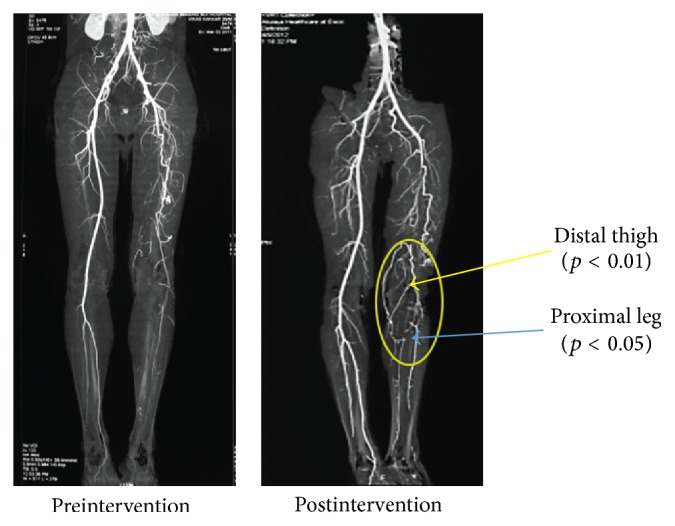
CT Angiography images showing qualitative analysis of degree of angiogenesis.

Computed Tomography (CT) Angiography, a diagnostic test that utilizes X-ray technology and 3-dimensional imaging of the blood vessels and surrounding tissues of the affected limb, was performed to determine the location and severity of artery narrowing or blockage and to measure the degree of angiogenesis from baseline. The CT Angiography (CTA) of the lower extremities reveals multiple focal points of stenosis with reduced collateral blood flow at baseline. CT Angiography was performed upon screening and at 12-month follow-up visit. A validated unique scoring system has been used to quantify the collateral vessels based on their size (collateral vessel grades 0 to 4) and collateral number (collateral vessel categories 0 to 3) in proximal, mid, and distal regions of the afflicted thigh and leg. The description and summary of the scoring system is given in Table 3. The CT Angiography grading and category results for the number of collaterals formed and the size of collaterals are summarized in Table 4. A statistically significant increase in the number of collateral vessels was observed in the distal thigh (*p* < 0.01) and proximal leg (*p* < 0.05) compared to baseline, while the size of collaterals was statistically increased in the distal thigh (*p* < 0.01) as compared to baseline. The results indicate that there is a slight to moderate increase in the capillary collateral size and capillary collateral numbers post-BMC treatment.  Figure 9 shows the CTA images of patient at baseline and 12 months, depicting the qualitative analysis of degree of angiogenesis.

## 4. Discussion

The critical limb ischemia-Stem Cell Therapy (CLI-STEM) feasibility, nonrandomized, open-label, single-arm, single-centre study was designed to evaluate the safety and preliminary effectiveness of harvesting and injecting intramuscularly autologous bone marrow cell concentrate in patients with nonreconstructable critical limb ischemia wherein each patient was treated with bone marrow derived progenitor cells harvested and processed using a rapid point-of-care device, Res-Q 60 BMC system. This feasibility study was intended to present a safety profile for a rapid point-of-care processing and delivery device and to lay the platform for a pivotal trial. The results from the study were significant, indicating the safety, feasibility, and plausible preliminary effectiveness of cellular therapy in patients with no-option CLI. In addition, our data suggests that the use of bone marrow derived cell product could potentially increase limb perfusion and improve claudication symptoms of limb ischemia, as our results demonstrated a significant increase in ABI, TcPO_2_ levels, and pain-free walking distance.

Ischemia is a potent stimulus to increase the oxygen delivery via a network of collateral vessels and to stimulate angiogenesis [34, 35], but this natural capability is impaired in CLI patients [36, 37]. Stimulation of vessel growth and/or remodelling has emerged as a new therapeutic option in patients with ischemic diseases. Different strategies of therapeutic angiogenesis, based on administration of recombinant growth factors such as, Hepatocyte Growth Factor (HGF), cells with induced angiogenic proteins (VEGF) and bone marrow derived, autologous or allogeneic, progenitor cell mixtures, have been proposed and currently investigated in human studies. Autologous bone marrow- (BM-) derived stem and progenitor cells have been identified as a potential new therapeutic option to induce therapeutic angiogenesis as the unfractionated mixture of bone marrow derived nucleated cells includes a significant number of differentiated cells that are thought to provide angiogenic cytokines as well as stem cells that become incorporated into collateral vessels by a process of neoangiogenesis [38]. The autologous BM cell (aBMC) concentrate is derived from the bone marrow aspirate by density gradient centrifugation and consists of (a) an acellular fraction comprised of autologous plasma and the cytokines and (b) a cellular fraction which is (i) reservoir of progenitor cells at several levels of maturity and multipotency and (ii) source of proangiogenic cells such as hematopoietic stem cells, mesenchymal progenitor cells, and endothelial progenitor cells. The biological components and their plausible roles in tissue repair and regeneration have made aBMC an attractive source of cells for therapeutic angiogenesis in the treatment of ischemic diseases [39].

A recent review by Compagna et al. showed that several studies have used cell therapy for no-option CLI patients and, despite some failures due to factors that invalidated the functionality of stem cells (i.e., diabetes), the results obtained have confirmed the beneficial effects of cell therapy in reducing the major amputation rate, improving distal perfusion, reducing rest pain and claudication pain, improving ABI, TcPO_2_, and walking distance, and overall improvement in the ischemic symptoms of CLI patients and their quality of life [40]. Furthermore, in the meta-analysis by Wang et al., where 31 published Randomized Controlled Trials (RCTs) and non-RCTs, having a total of 1,214 patients, were evaluated, and advantageous effects of autologous bone marrow cell therapy was reported. In the selected studies, the majority of severe adverse events (SAEs) were associated with hospitalization for disease process-related complications and not related to cell therapy. The Ankle Brachial Index (ABI) and pain scores showed significant improvement after cell therapy in both non-RCT and RCT groups. Most importantly, the long term (>1 year) clinical trials demonstrated that the amputation-free survival (AFS) rate improved after therapy with bone marrow cells [28]. Benoit et al. evaluated RCTs involving bone marrow derived stem and progenitor cells (*n* = 295) and found that the amputation rates between the control arms and treatment arms were statistically significant (25.4% versus 14.8%  *p* = 0.02) demonstrating that bone marrow derived cells do improve outcomes in CLI patients [41]. In a review of 57 early-phase clinical trials, the safety and feasibility of autologous bone marrow cell transplantation was reported in 1667 human subjects [42]. Our results were in concurrence with previously published studies and we demonstrated the potential safety and feasibility of autologous bone marrow cell concentrate in no-option CLI patients.

In addition to the type of cell used for treatment, the route of administration for cell therapy has also been a major subject of debate in previous clinical trials [43]. The two main routes of administration that have been used in studies are intramuscular (IM) and intra-arterial (IA) route of delivery. Intramuscular delivery of bone marrow derived progenitor cells allows direct placement of therapeutic cells into the ischemic tissue where neovascularization is required. However, on the other hand, intra-arterial delivery may direct the cells to viable peri-ischemic zones, where there is sufficient nutrient supply to support cell proliferation and differentiation [12]. Previous studies showed intramuscular* (IM)* and intra-arterial* (IA)* injection or a combination of both has yielded promising results in human PAD [44, 45]. To date, majority of the cell therapy clinical trials have used intramuscular infusion of therapeutic cells and it seems to be a safe and effective procedure, confirming the findings of our study, where intramuscular delivery of autologous bone marrow cell concentrate was used as the route of administration. We propose that, on intramuscular delivery, plasma present in the bone marrow concentrate helps in distribution of angiogenic cells and cytokines along the muscle fibres, and fibrinogen, present in plasma, creates a net that catches the injected platelets and cells across the ischemic tissue, which upon activation stimulate the inflammatory cascade and proceeds towards healing [46].

The main finding of our study was that BMC therapy significantly facilitated the reduction of major limb amputations at 12-month follow-up, demonstrating a 70.6% amputation-free survival rate in no-option CLI patients. Previous uncontrolled studies using intramuscular injection of BMMNC or blood-derived mononuclear cells reported 6-month limb salvage rates of 71% [47] and 59% [17]. Thus autologous BM-derived cell therapy seems to be a relatively safe and feasible treatment option with no or minimal adverse effects. Enrolled CLI patients were either suffering from atherosclerotic PAD or Thromboangiitis Obliterans (TAO), also known as Buerger's disease. CLI is the most severe form of atherosclerotic PAD affecting majority of the patients with PVD, while 20–40% of these patients are estimated to have Buerger's disease which is more common in younger population especially in those who smoke a crude and high concentrated form of tobacco leaves [17, 48]. TAO is the progressive inflammation and thrombophlebitis in small and medium sized arteries that causes severe pain at rest in the limb and may lead to gangrene. It has been reported in previous studies that Buerger's disease patients have lower mortality and morbidity rates as compared to atherosclerotic PAD patients as its pathophysiology is not associated with cardiovascular risk factors [26]. In our study, better clinical outcomes and lower mortality rates were observed in Buerger's disease patients. No mortality or morbidity was observed in Buerger's patients and none of them underwent any amputation (minor or major) during the 12-month follow-up period. The amputation-free survival rates in atherosclerotic PAD and Buerger's patients were 46.5% and 100%, respectively, indicating a significant prognosis in Buerger's disease patients. Our study results are in concurrence with previously published studies [26, 49]. Although there is no clear justification on the mechanism of action for superior results in Buerger's disease patients, however the burden of cardiovascular risk factors is considerably less and, therefore, less dysfunction of progenitor cells and less violent systemic milieu are present in this patient population which could result in superior results in Buerger's disease patients. Furthermore, our study had a very small sample size of Buerger's patients to definitely conclude our statement and, therefore, further studies with larger patient population are required to establish the difference in treatment efficacy between Buerger's disease and atherosclerotic PAD patients using bone marrow cell therapy. In our study, 3 major limb amputations were reported in the atherosclerotic PAD patient population, at four-month, 6-month, and 12-month post-BMC implantation, and the amputations were testified as being a normal progression of the disease in response to clinical deterioration or because of lack of improvement. The study results were very promising, and the study achieved limb salvage of significant number of patients' post-BMC treatment and our results were similar to the findings of other studies where CLI patients have been treated with bone marrow derived mononuclear cells [3, 15, 16].

In contrast, a recent meta-analysis by Peeters Weem et al. of randomized placebo-controlled trials in CLI patients has shown no significant differences in amputation rates or amputation-free survival between cell therapy treated and placebo treated patients [50]. Further, the JUVENTAS-trial, a double-blinded, placebo-controlled study with 160 patients, showed no significant difference in amputation rates at 6 months between the bone marrow treated (19%) and placebo treated (13%) groups [51]. Similarly, PROVASA study which treated 40 patients with CLI either with intra-arterial injections of BMMNC or placebo control in a multicentred, phase II, double-blind, placebo-controlled trial showed no significant difference in the limb salvage and amputation-free survival rates between the two groups [52]. Additionally, few more pilot studies with small sample size did not show any significant difference in the cell therapy treatment and placebo control groups in CLI patients [53, 54]. Although, these published studies contradict the therapeutic effectiveness of bone marrow cell therapy in reducing amputation rates as compared to placebo control, they are relatively small trials with small sample size primarily designed for safety analysis, and larger double-blinded, placebo-controlled studies are required to prove the therapeutic effectiveness of cell therapy.

Furthermore, BMC therapy has shown to have beneficial effect on other physiological and anatomical parameters, including ulcer healing, ABI, TcPO_2_, pain-free walking distance, and the rest pain score. Considering the perfusion analysis, we evaluated the improvement in limb perfusion by reduced ischemic pain (assessed by VAS), improved walking distance, signs of wound healing, and improved ABI and TcPO_2_ measurements. These indicators are more objective as compared to the endpoints used by other studies like, numbness, cold sensation, and pain [27, 55]. In our study, Ankle Brachial Index (ABI) significantly increased 1 month after BMC treatment and reached a peak value at 6 months posttreatment when compared to baseline. Thereafter, ABI gradually decreased until 12-month follow-up; however it did not reach the baseline level. The results for Transcutaneous Oxygen Pressure (TcPO_2_) showed a significant increase by one month and reached a peak at 12-month follow-up post-BMC implantation. The change in ABI and TcPO_2_ correlates with improvements in the clinical endpoints including ulcer healing and rest pain reduction. Our data on change in ABI following bone marrow derived cell therapy is in concurrence with previously published studies [14, 21, 29, 48, 56–59]. In contradiction to the above results, few studies have reported that BMC infusion did not alter ABI but accelerated ulcer healing and rest pain reduction in PAD patients [52]. The PROVASA trial used ABI assessment as primary endpoint for their study; however their study results concluded that choice of changes in ABI as a primary endpoint was the major drawback of this study as change in ABI values did not correlate with improvements in the clinically most relevant therapeutic goals of treatment, that is, ulcer healing and limb salvage [52, 60]. A similar discordance has been observed between clinical endpoints and functional endpoints in a gene therapy trial for therapeutic angiogenesis in CLI patients [61]. In contrast, our study data showed a strong correlation between the improvement of TcPO_2_ to reduced rate of amputation by fitting a Cox proportional hazard model and thus TcPO_2_ can safely predict the amputation events. A similar correlation has been shown by other investigators where a predictive value of Transcutaneous Oxygen Tension in diabetic patients for above-the-ankle amputation with critical limb ischemia was shown [26, 62].

Autologous bone marrow derived mononuclear cells has been shown to increase pain-free walking distance significantly in patients with severe Peripheral Arterial Disease (PAD) and critical limb ischemia at 12-month follow-up [63]. As mentioned in the literature we observed that 100% of the enrolled patients in our study showed improvement in rest pain, while intermittent claudication pain improved in 90% of the patients. These results were superior to other studies, where Gabr et al. reported improvement in rest pain in 55% of the patients, Chung and associates reported 88.2% improvement, and Tateishi-Yuyama et al. reported 50% regression of rest pain [21, 27, 55]. Furthermore, the number of subjects with potential to walk and perform the 6-minute walk test significantly increased post-BMC infusion and the results showed that 65% of the patients were able to walk a mean distance of 210.0 meters in 6 minutes at 12-month follow-up as compared to 14% of the patients who were able to walk at baseline. There are few trials that have used walking times or distances measured by standardized treadmill protocols or a 6-minute walk test as a primary endpoint or even a secondary efficacy endpoint. The 6-min walk test measures the maximal distance walked after 6 min, regardless of whether the patient stops to rest or not; thus, this test can be used to evaluate patients with severe claudication, ischemic rest pain, or limited tissue loss [64]. Like other functional measures this endpoint was felt to be clinically meaningful and pragmatically useful, as it both characterizes a patient's limitation at baseline and the response to treatment.

Under normal/healthy conditions the response to gradually progressing limb ischemia involves the promotion of angiogenesis and arteriogenesis in an attempt to increase the blood supply to the affected limb. Vascular remodelling, inflammation, and apoptotic pathways are implicated in the ischemic response and these may in part contribute to the resolution of tissue damage. In brief, tissue healing process after limb ischemia can be divided into three phases: (i) thrombus formation-induced hypoxia, leading to hypoperfusion and metabolic dysregulation where the damaged cells undergo apoptosis and necrosis; (ii) inflammatory cells, neutrophil, monocytes, and lymphocytes, home to necrotic tissue for removal of dead cells; and (iii) remodelling and regeneration eventually leading to formation of a scar tissue [65]. In patients with ischemic disease, the cellular and molecular mechanisms involved in the activation of vessel growth and vascular remodelling are markedly impaired by the deleterious microenvironment characterized by fibrosis, inflammation, and tissue hypoperfusion.

Broadly speaking, vascular regeneration includes the restoration of normal vascular function and structure, the reversal of vascular senescence, and the growth of new blood vessels [66]. This includes a wide range of processes such as the distribution of blood flow via the formation of new collateral networks; the response of newly generated vessels to hemodynamic, humoral, and local tissue factors; the modulation of the immune response and the trafficking of circulating cells; and the permeation of nutrients and macromolecules through the microvasculature, which can in turn have trophic effects on blood fluidity and hemostasis [42, 67]. Neovascularization is a process that involves the formation of collateral vessels from preexisting occluded vessels by migration, proliferation, and differentiation of progenitor cells and the interplay between growth factors and cytokines. The process of neovascularization comprises three distinct phenomena: (i) vasculogenesis, (ii) angiogenesis, and (iii) arteriogenesis [68]. Neovascularization, in response to local tissue ischemia, occurs not only by migration and proliferation of resident mature endothelial cells, but also by involving bone marrow- (BM-) derived endothelial progenitor cells (EPC) [69]. In response to hypoxia, the local production of chemokines and growth factors such as stromal cell-derived factor-1*α* (SDF-1*α*) and Vascular Endothelial Growth Factor (VEGF) are upregulated, leading to elevated blood levels of these substances. In the BM microenvironment these factors induce the release and activation of matrix metalloproteinases (MMPs) causing EPCs, which are positive for the SDF-1*α* receptor CXCR4 and VEGF receptor 2 (VEGFR-2, KDR), to move to the circulation [70]. EPCs subsequently contribute to neovascularization, either by physical incorporation into the endothelial layer or by excretion of paracrine factors that stimulate proliferation of resident endothelial cells [69], the latter being likely the paramount mechanism [71, 72] occurring in concert with other circulating cells such as monocytes [73, 74].

**Figure 10:**
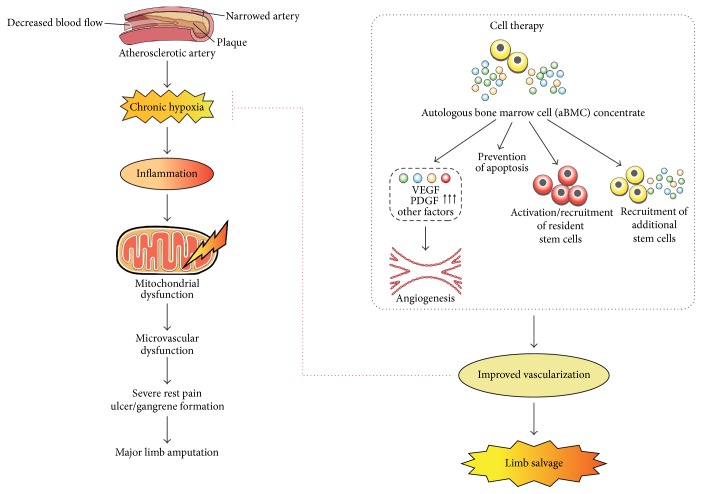
Diagrammatical representation of the mechanism of action of cell therapy in CLI patients.

The precise mechanism of action for injected BMC in CLI patients is yet not well established. It is unclear if the injected cells are directly involved in the angiogenic process or act as cytokine factories releasing multiple different growth factors that have a paracrine effect. Several presumed mechanisms of activity of cell therapy in CLI have been reported by previous studies; these include (i) increased tissue vascularity; (ii) increased tissue microperfusion at the capillary level; (iii) remodelling of fibrotic tissues to allow new capillary growth or increase interstitial fluid flow; (iv) improved mitochondriopathy; and (v) modulation of the inflammatory response program from tissue destruction to wound healing. Since the possibility of a combination of these mechanisms in improving the ischemic condition may be synergistic, the complex mixture of bone marrow concentrate which includes hematopoietic stem/progenitor cells, mesenchymal stem/progenitor cells, endothelial progenitor cells, and inflammatory cell precursors may be important in their therapeutic function. For instance, the presence of CD14^+^ macrophages may promote the dissolution of extracellular matrix in the perivascular interstitial space and thus enhances vessel sprouting by endothelial precursor cells [18, 29]. In principle the increased blood flow is achieved by increasing the number of blood vessels or collaterals that supply blood to ischemic tissue. Implantation of autologous bone marrow cells is demonstrated to be an effective and feasible technique of inducing therapeutic angiogenesis in both clinical and experimental studies. However, the angiogenic potency might differ among cell fractions of bone marrow cells and which of these play a key role remains unclear [27, 55].  Figure 10 gives a diagrammatic representation of the events that follow during ischemic disease and plausible mechanism of action of injected bone marrow progenitor cells in increasing vascularity and limb salvage.

In our study, an independent blinded core lab is evaluated for quantitative and qualitative changes in the CT angiographies of the afflicted limb at baseline and 12 months. CTA imaging of the thigh and calf muscle region showed differences in terms of visible collateral formation in distal thigh and proximal leg after aBMC treatment at 12 months when compared to baseline indicative of vasculogenesis. The quantitative grading of the collaterals showed a significant difference in the mean collateral numbers in distal thigh and proximal leg region at 12 months post-BMC treatment compared to baseline, while in contrast the mean collateral size significantly increased only in distal thigh region at 12 months post-BMC treatment. Similar type of results has been shown by Yoon et al., where they witnessed increased capillary and arteriolar vessel density when BMMNC are administered in an experimental model of myocardial infarction [75]. Although not much literature is available on the use of CTA for quantitative evaluation of collateral vessels and most of the published studies have used CTA for confirming enhancement in peripheral collateral circulation distal to arterial occlusion [76–78], it is also evident from published data that the visualization of collateral vessels is more extensive on Digital Subtraction Angiography (DSA) as compared to CTA [76]. But, due to the limited available data, these assertions may not be true. Furthermore, Qenawy et al. demonstrated significant difference in the visualization and quantification of collateral vessels by multidimensional CTA as compared to DSA [77]. CTA serves as a successful imaging modality for demonstrating peripheral arterial occlusion or stenosis. It can also be useful in assessing the presence of collaterals that could potentially serve as an important indicator for qualitative and quantitative prognosis of the treatment.

The infused cell dose plays a pivotal role in the effectiveness of cellular therapy. The point-of-care system (SurgWerks-CLI Kit) used in this study for cell preparation demonstrated several advantages as it is a rapid, single sitting procedure that reduces time, cost, and manual intervention for processing and injecting bone marrow cell concentrate in the ischemic limb of the CLI patients and the complete procedure was completed within 90 minutes in the operation theatre. Furthermore, the system was capable of achieving mean total nucleated cell counts of 8.04 × 10^8^ (±3.66) from 120 mL of marrow aspirate, out of which the mean viable CD34^+^ cells were 4.06 × 10^6^ (±3.82) and the mean total colony forming units for CD34^+^ were 101.10 × 10^4^ (±113.61). These numbers are comparable to those achieved by manual Ficoll based protocols that require 500 mL of bone marrow aspirate. The smaller volume of marrow leads to shorter marrow harvesting times, less anaesthesia risk, and less anemia [79].

## 5. Conclusion

Administration of autologous bone marrow mononuclear cells' injection is easy, inexpensive, and safe, with a definite ameliorating effect on limb ischemia. However, specification of the target cell population, route of administration, and dose escalation need larger sample size and controlled studies. Our phase Ib/feasibility study demonstrated that the SurgWerks-CLI Kit including the Res-Q 60 BMC system is safe and feasible for harvesting, processing, and intramuscularly injecting aBMC in the ischemic limbs and the preliminary efficacy results indicated that aBMC therapy potentially improved major amputation-free survival rate, 6-minute walking distance, Ankle Brachial Index, TcPO_2_, rest pain, and intermittent claudication pain scores in no-option CLI patients. The major study limitations were (i) it was a single-arm study, (ii) small sample size that was designed for safety analysis, and (iii) it was a single-centre study. Therefore, based on these promising results and considering the study limitations, we have initiated a phase III/pivotal, multicentre, randomized controlled, double-blinded study with larger sample size in no-option CLI patients. In conclusion, cellular therapy is well tolerated and offers rising hope for patients with critical limb ischemia.

## Figures and Tables

**Table 1:** Clinical characteristics of the patients enrolled in the study at baseline.

Baseline characteristics of enrolled patients	Value (*n* = 17)	%
Age (mean ± SD)	48.8 ± 13.07	
Gender		
Male	15	88.2
Female	2	11.8
Affected limb		
Left	9	52.9
Right	8	47.1
Peripheral Arterial Disease (PAD)	13	76.5
Buerger's disease	4	23.5
Rutherford classification (grade 5)	17	100
Fontaine classification (grade IV)	17	100
Smoking status		
Smoker	15	88.2
Nonsmoker	2	11.8
Comorbidities		
Diabetes	3	17.6
Hypertension	3	17.6
Coronary Artery Disease (CAD)	3	17.6
Thrombocytopenia	0	0

**Table 2:** Cell product characterization.

	Preprocessed (mean ± SD)	Postprocessed (mean ± SD)
Cellularity^*∗*^		
TNCC (×10^8^)	2.64 ± 1.29	8.04 ± 3.65
MNCC (×10^8^)	0.59 ± 0.26	2.16 ± 1.02
CD34+ve (×10^6^)	1.33 ± 2.09	4.05 ± 3.81
Cell viability (%)	72.02 ± 19.73	88.94 ± 5.54
Potency		
Total CFUs (×10^4^)	ND	101.1 ± 113.61
Sterility	No growth	No growth

^*∗*^Mean fold increase in postprocessed sample is about 3x.

**Table 3:** Definition of the classification system used to quantify the collateral vessel number and size.

Category	Number of collaterals^a^
Category 0	0 collateral vessels
Category 1	1–3 collateral vessels
Category 2	4–7 collateral vessels
Category 3	≥8 collateral vessels

Collateral vessel grade	Size of collateral vessels

Grade 0	0 collateral vessels
Grade 1	≤5 small
Grade 2	>5
Grade 3	≤5 large ± small
Grade 4	>5 large ± small

^a^Small collaterals occupy <25% of the length of the imaged thigh and <50% of the diameter of the superficial femoral artery. Large collaterals occupy >25% of the length of the imaged thigh and ≥50% of the diameter of the superficial femoral artery.

**Table 4:** Summary of CT Angiography category and grading results.

Summary of CT Angio peripheral extremities: size (grade) and number (category) of collaterals vessels										
	Collateral vessel grade [*n* (%)]^*∗*^				Mean (SD)	Collateral vessel category [*n* (%)]^*∗∗*^				Mean (SD)
	Grade 0	Grade 1	Grade 2	Grade 3		Category 0	Category 1	Category 2	Category 3	

Proximal thigh										
Preintervention	9 (64.3)	1 (7.1)	0	2 (14.3)	0.58 (1.165)	9 (64.3)	1 (7.1)	2 (14.3)	0	0.42 (0.793)
Postintervention	7 (50.0)	1 (7.1)	1 (7.1)	3 (21.4)	1.00 (1.348)	7 (50.0)	2 (14.3)	2 (14.3)	1 (7.1)	0.75 (1.055)
Mid thigh										
Preintervention	7 (50.0)	3 (21.4)	2 (14.3)	0	0.58 (0.793)	7 (50.0)	3 (21.4)	2 (14.3)	0	0.58 (0.793)
Postintervention	5 (35.7)	2 (14.3)	3 (21.4)	2 (14.3)	1.17 (1.193)	5 (35.7)	3 (21.4)	0	4 (28.6)	1.25 (1.357)^#^
Distal thigh										
Preintervention	8 (57.1)	4 (28.6)	0	0	0.33 (0.492)	8 (57.1)	3 (21.4)	1 (7.1)	0	0.42 (0.669)
Postintervention	4 (28.6)	2 (14.3)	4 (28.6)	2 (14.3)	1.33 (1.155)^#^	4 (28.6)	3 (21.4)	1 (7.1)	4 (28.6)	1.42 (1.311)^#^
Proximal leg										
Preintervention	10 (71.4)	2 (14.3)	0	0	0.17 (0.389)	10 (71.4)	1 (7.1)	1 (7.1)	0	0.25 (0.622)
Postintervention	5 (35.7)	3 (21.4)	2 (14.3)	2 (14.3)	1.08 (1.165)^#^	5 (35.7)	2 (14.3)	4 (28.6)	1 (7.1)	1.08 (1.084)^#^
Mid leg										
Preintervention	10 (71.4)	1 (7.1)	1 (7.1)	0	0.25 (0.622)	10 (71.4)	0	2 (14.3)	0	0.33 (0.778)
Postintervention	8 (57.1)	3 (21.4)	1 (7.1)	0	0.42 (0.669)	8 (57.1)	1 (7.1)	2 (14.3)	1 (7.1)	0.67 (1.073)
Distal leg										
Preintervention	10 (71.4)	1 (7.1)	1 (7.1)	0	0.25 (0.622)	10 (71.4)	0	1 (7.1)	1 (7.1)	0.42 (0.996)
Postintervention	10 (71.4)	1 (7.1)	1 (7.1)	0	0.25 (0.622)	10 (71.4)	0	1 (7.1)	1 (7.1)	0.42 (0.996)

^#^ *p* < 0.05: values are statistically significant compared to preintervention.

^*∗*^Collateral vessel grading is a system used to quantify the size of the collateral vessels formed as follows:

Grade 0: no collateral vessels.

Grade 1: ≤5 small.

Grade 2: >5 small.

Grade 3: ≤5 large ± small.

Grade 4: >5 large ± small.

^*∗∗*^Collateral vessel category is a system used to quantify the number of collateral vessels present as follows:

Category 0: no collateral vessels.

Category 1: 1–3 collateral vessels.

Category 2: 4–7 collateral vessels.

Category 3: ≥8 collateral vessels.
